# DNASU plasmid and PSI:Biology-Materials repositories: resources to accelerate biological research

**DOI:** 10.1093/nar/gkt1060

**Published:** 2013-11-12

**Authors:** Catherine Y. Seiler, Jin G. Park, Amit Sharma, Preston Hunter, Padmini Surapaneni, Casey Sedillo, James Field, Rhys Algar, Andrea Price, Jason Steel, Andrea Throop, Michael Fiacco, Joshua LaBaer

**Affiliations:** ^1^Virginia G. Piper Center for Personalized Diagnostics, Biodesign Institute, Arizona State University, 1001 S. McAllister Dr. Tempe, AZ 85287-6401, USA and ^2^LabGenius, 20-22 Bedford Row, London, WC1R 4JS, UK

## Abstract

The mission of the DNASU Plasmid Repository is to accelerate research by providing high-quality, annotated plasmid samples and online plasmid resources to the research community through the curated DNASU database, website and repository (http://dnasu.asu.edu or http://dnasu.org). The collection includes plasmids from grant-funded, high-throughput cloning projects performed in our laboratory, plasmids from external researchers, and large collections from consortia such as the ORFeome Collaboration and the NIGMS-funded Protein Structure Initiative: Biology (PSI:Biology). Through DNASU, researchers can search for and access detailed information about each plasmid such as the full length gene insert sequence, vector information, associated publications, and links to external resources that provide additional protein annotations and experimental protocols. Plasmids can be requested directly through the DNASU website. DNASU and the PSI:Biology-Materials Repositories were previously described in the 2010 NAR Database Issue (Cormier, C.Y., Mohr, S.E., Zuo, D., Hu, Y., Rolfs, A., Kramer, J., Taycher, E., Kelley, F., Fiacco, M., Turnbull, G. *et al.* (2010) Protein Structure Initiative Material Repository: an open shared public resource of structural genomics plasmids for the biological community. *Nucleic Acids Res.*, 38, D743–D749.). In this update we will describe the plasmid collection and highlight the new features in the website redesign, including new browse/search options, plasmid annotations and a dynamic vector mapping feature that was developed in collaboration with LabGenius. Overall, these plasmid resources continue to enable research with the goal of elucidating the role of proteins in both normal biological processes and disease.

## INTRODUCTION

Plasmids form a cornerstone in modern molecular and structural biology research laboratories. Considerable time, expertise and resources are expended cloning genes into the appropriate vector to express recombinant proteins for both *in vivo* functional studies or to produce purified proteins for biochemical studies. Many laboratories that work with limited budgets or have limited experience with the molecular biology techniques required for gene cloning create significant demand for pre-made plasmids. Furthermore, after laboratories have created and used plasmids for experiments and referenced them in publications, they are frequently lost or forgotten in the back of freezers, often known only to the students or postdocs who have since left the laboratory, thus losing a physical resource that would be valuable to the broader scientific community.

Commercial, nonprofit and laboratory groups have recognized the need to archive plasmids and make them available to the research community [selected references (1–7)]. These plasmid collections often focus on a particular theme or specialized collection. For example, the NIAID-funded BEI resources focuses on genes from organisms that cause infectious disease, the Drosophila Genomics Resource Center provides cell lines and plasmids from one model organism, *Drosophila melanogaster*, the nonprofit Addgene primarily distributes plasmids submitted by individual researchers after publication, and the for-profit company Invitrogen focuses on large collections based on a specific technology (such as Gateway® or shRNA expression) or an organism (such as human open reading frames or yeast BACS). All have the goal of providing plasmids to researchers, though they differ widely in the plasmids that they distribute as well as the costs, annotations, website accessibility and customer support.

The DNASU plasmid repository was established at the Biodesign Institute at Arizona State University as a nonprofit service core with the goal of providing sequence-verified, highly-annotated plasmids to researchers though an easy-to-use and accessible website (http://dnasu.org). In addition to functioning as a repository for plasmids that are created within our center, DNASU also stores and distributes plasmids created by individual external researchers and by collaborative groups of investigators like the ORFeome Collaboration (8,9) and the Protein Structure Initiative:Biology (PSI:Biology) (1), a multicenter biologically focused structural biology effort. DNASU focuses both on storing valuable plasmids that researchers and consortiums have created and on distributing these highly-annotated plasmids and plasmid collections to researchers worldwide. The goal is for researchers to avoid the time and effort needed to reclone genes so that they can focus immediately on their experiments, thus accelerating the pace of discovery.

## DATABASE DESCRIPTION

### Plasmid collection overview

Since the first report in 2010 (5,10), the number of plasmids stored and distributed through DNASU has nearly doubled to 198 000 plasmids containing gene inserts (e.g. cDNA, genomic DNA, intergenic regulatory element, etc.) from over 700 species. Over half of these plasmids contain genes from eukaryotic organisms, 45% from prokaryotes, with the remainder from archaea, viruses, and metagenome cloning projects. Over 74 000 of these plasmids contain human genes, representing ∼13 000 unique human genes.

As the PSI:Biology-Materials Repository (PSI:Biology-MR), 81 000 protein expression plasmids, ∼40% of the collection, were created and deposited by PSI:Biology centers. Of these, ∼3300 were used to solve 3D protein structures that have been deposited in the Protein Data Bank (PDB). Each of these plasmids has been fully sequence-verified at the PSI:Biology-MR and links to additional annotations, experimental protocols and contact information for the depositing laboratories to facilitate in the expression and purification of individual proteins.

To enhance functional annotation of the plasmids, we recently scanned protein sequences of the entire DNASU collection for 1067 protein domains by HMMER2 (http://hmmer.janelia.org/) and the SMART HMM (hidden Markov model) motifs (http://smart.embl-heidelberg.de/) (11) with an *E* value threshold of 0.05. Based on this analysis, 978 domains were found in the collection. As an example, across the human proteome, the Gateway® donor collection covers 45% to 90% of the 34 most abundant domains that are found in more than 100 proteins, including RHO GTPases (90% coverage), protein kinases (88%), the protein–protein interaction BTB domain (82%), the phosphotyrosine–binding SH2 domain (80%) and the multifunctional WD40 domain (74%) (Table 1). The domain information has been implemented into DNASU as a part of the new browse feature (described in more detail below).

**Table 1.** DNASU plasmid coverage of genes with SMART domains that are found in at least 100 human proteins

Domain	Description	# Genes in human genome	# Genes in gateway donor vectors in DNASU	Coverage (%)
RHO	Rho (Ras homology) subfamily of Ras-like small GTPases	135	121	89.6
RAB	Rab subfamily of small GTPases	160	142	88.8
RAN	Ran /TC4 subfamily of small GTPases	107	94	87.9
S_TKc	Serine/Threonine protein kinases, catalytic domain	484	425	87.8
TyrKc	Tyrosine kinase, catalytic domain	468	408	87.2
RAS	Ras subfamily of RAS small GTPases	146	126	86.3
Tryp_SPc	Trypsin-like serine protease	116	96	82.8
BTB	Broad-Complex, Tramtrack and Bric a brac	178	145	81.5
SH2	Src homology 2 domains	108	86	79.6
IG	Immunoglobulin	432	337	78.0
HLH	Helix–loop–helix domain	117	88	75.2
RRM	RNA recognition motif	233	172	73.8
WD40	WD40 repeats	253	186	73.5
SH3	Src homology 3 domains	212	154	72.6
EFh	EF-hand, calcium-binding motif	154	106	68.8
TPR	Tetratricopeptide repeats	106	72	67.9
HOX	Homeodomain	254	171	67.3
ZnF_C2H2	Zinc finger	747	487	65.2
KRAB	Krueppel-associated box	379	243	64.1
HELICc	Helicase superfamily C-terminal domain	108	68	63.0
CA	Cadherin repeats	116	72	62.1
PDZ	Domain present in PSD-95, Dlg and ZO-1/2	148	91	61.5
PH	Pleckstrin homology domain	271	166	61.3
IGc2	Immunoglobulin C-2 Type	326	196	60.1
ANK	Ankyrin repeats	235	140	59.6
DEXDc	DEAD-like helicases superfamily	124	73	58.9
IGv	Immunoglobulin V-Type	177	104	58.8
RING	Ring finger	301	174	57.8
C2	Protein kinase C conserved region 2 (CalB)	130	75	57.7
FN3	Fibronectin type 3 domain	163	89	54.6
LRR_TYP	Leucine-rich repeats, typical (most populated) subfamily	158	86	54.4
AAA	ATPases associated with a variety of cellular activities	184	91	49.5
EGF_CA	Calcium-binding EGF-like domain	153	72	47.1
EGF	Epidermal growth factor-like domain	176	80	45.5

Only the plasmids in Gateway donor vectors were counted.

To expedite systems biology and other high-throughput studies, DNASU also provides preorganized plasmid collections to researchers at a reduced recharge cost. DNASU distributes whole-genome collections from yeast and various pathogens as well as specialized collections of human genes, such as kinases or genes involved in breast cancer (listed here http://dnasu.org/DNASU/Browse.do?tab=0).

#### Vectors

Genes in DNASU are cloned into vectors for numerous downstream applications including bacterial, yeast, mammalian, baculovirus or cell-free expression. In addition to plasmids containing gene inserts, the repository distributes 138 cloning or expression empty vectors (Table 2). These vectors, especially those created by the PSI centers, have been optimized for high-level protein expression and purification in bacteria, wheat germ extract (12,13), mycoplasma (14) or yeast (15) and use different tag configurations and protease cleavage sites to accommodate a variety of experimental designs. Many of these vectors also take advantage of fast ligation-independent technologies such as Gateway® (16), Ligation Independent Cloning (LIC) [representative publications (17,18)] and Polymerase Incomplete Primer Extension (PIPE) (19), allowing for rapid and easy cloning of a gene insert into the vector of interest. Vector annotations on the DNASU website often include links to the PSI:Biology Structural Biology Knowledgebase (SBKB) Technology Portal (http://technology.lbl.gov/) (20), which provides detailed descriptions of the cloning or expression technology. Details and publications about the most common cloning methods used for DNASU plasmids can also be found in the new Resources section (http://dnasu.org/DNASU/Resources/index.jsp).

**Table 2.** Number of empty vectors available in DNASU categorized by expression system

Type of empty vector	# Empty vectors
Bacterial expression	
Expression	71
Coexpression	13
Increased solubility	3
Mycobacterial coexpression	12
Yeast expression	8
Mammalian expression	8
Cell-free expression	20
Screening/Cloning	3
TOTAL	138

#### Downloading DNASU data

Several options to download plasmid data are available to researchers who wish to analyze the entire collection using their own informatics tools, to sort search results or to track all of the plasmids they have ordered. On the BLAST search page (http://dnasu.org/DNASU/SearchOptions.do?tab=4), data for DNASU plasmids are available as FASTA-formatted files containing either all nucleotide or amino acid sequences with their associated plasmid IDs and vector names. These files are regularly updated when new plasmids are added to the repository. Individual search results can also be downloaded as an Excel file that includes the gene name, vector, growth conditions, gene insert sequence or collection information. Researchers may also download their ordering history and the list of plasmids in each order to track and find information on the plasmids they have requested from DNASU.

## NEW AND ONGOING DEVELOPMENT

### Search options

DNASU aims to facilitate research, and to accomplish this goal, we strive to help researchers find what they are looking for on the website quickly and easily. To this end, DNASU’s five search options have been updated and six new browse features were created to provide flexibility in finding plasmids within the database. A key contribution has been to put search capabilities where researchers need them most. Thus, in the website redesign, a search box was added at the top of each page that will query the collection by any gene name or plasmid identifier. Since implementation, over one third of all searches are initiated through this universally-available search box.

The five standard searches (http://dnasu.org/DNASU/SearchOptions.do) [described in detail in (1,10)] are now presented in individual tabs on a single page allowing researchers to see and access all available search options quickly without switching web pages. The Advanced Search, PSI:Biology Search and Vector Search provide searches by gene or vector name, author name, species, PDB ID, Target Track ID (for PSI plasmids), protein expression data (if available) or vector characteristics such as appended polypeptide tags, selectable markers or protein expression systems. Both the Plasmid ID and BLAST Searches allow a researcher to search the database with multiple queries simultaneously. The Plasmid ID Search accepts up to 50 mixed identifiers, including DNASU plasmid IDs, gene names and gene accession numbers, and the BLAST Search accepts a FASTA file containing thousands of sequence queries.

Originally these search options were designed to perform optimally when querying a small database with several thousand entries. As the DNASU database has grown, the searches were optimized by introducing temporary lookup tables for every query. These lookup tables reduced the multiple database calls that fetched each detail for each clone, which significantly reduces the time it takes to perform large searches. After this optimization, searches with up to 20 different queries now average 2–3 s from clicking ‘search’ to receiving results.

### Browse options

As DNASU has accumulated a large number of plasmids, researchers may want to see all plasmids with a specific feature of interest or available predefined collections. To help researchers more efficiently explore the collection, find what they are looking for, or find something new, we have created categorized lists of plasmids presented in dynamic, easy-to-navigate browse features (http://dnasu.org/DNASU/Browse.do?tab=0). These six new browse options display preconfigured lists of annotations or information pulled directly from the database that summarize the materials available in DNASU, which provide a starting point to find plasmids based on particular criteria, such as species, protein domain or vector tag. The six browse features described below were chosen based on what we have observed researchers commonly search for, and designed in a visual format for easier use compared with a standard search feature.

**Figure 1.**
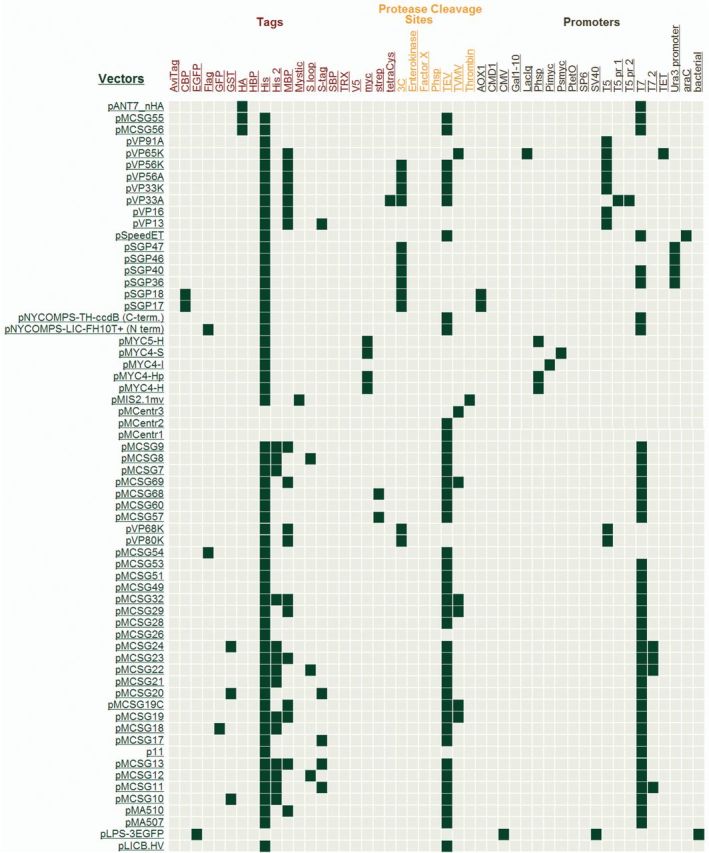
Screen shot of the dynamic vector browse feature. Clicking on any of the headings (tag, protease cleavage site or promoter) will sort the list to cluster the vectors by that feature dynamically. Clicking on the vector name will link to vector details pages and ordering information.

1. *Collections.* Browse all available preorganized collections. These collections include a set of human kinases, the ORFeome V8.1 collection both in a Gateway® entry vector and lentiviral expression vector (9), and whole genome collections from yeast and a variety of pathogens. In addition, we generated several collections that are based on widely studied protein families, such as kinases and phosphatases. These collections are stored on premade 96-well plates and can be requested at a discounted price.
2. *Biological function.* Browse for sets of plasmids with a particular function or feature, for example, all glycoenzymes, membrane proteins or G protein-coupled receptors. In order for researchers to obtain plasmid sets with specific biological functions, all clones were annotated for protein domains, and we implemented a plasmid browse function based on these domain names. This provides a powerful tool, allowing researchers to search the collection for plasmids representing a family of proteins that share specific domains or specific cellular functions (e.g. metabolic enzymes, kinases, DNA-binding proteins and membrane receptors) in a particular species (Table 1) or across many species.
3. *Vector.* Browse all empty vectors by characteristics such as polypeptide tags, protease cleavage sites and promoters (Figure 1). By clicking on a header element, this interactive table can be sorted instantly by vector name or by feature, creating a clustered table of empty vectors with, for example, a His tag. Clicking on the name of the empty vector will bring the researcher directly to the annotated vector features and ordering information.
4. *Author.* Browse alphabetically by the name of the person, laboratory, institution or consortium that created the plasmid. The output of this browse is a list of all plasmids created by that author.
5. *Species.* Browse by species name or type (e.g. bacteria, eukaryotes, archaea or viruses). This species list has been carefully annotated in the database using a controlled vocabulary based on the NCBI taxonomy database (http://www.ncbi.nlm.nih.gov/Taxonomy/taxonomyhome.html/), so that all plasmids that contain genes from subspecies, strains or variants of the species (e.g. *Mycoplasma pneumoniae M129*) will appear in the search by representative species names (e.g. *M. pneumonia*). The output of this browse function is a list of all plasmids containing inserts from that species.
6. *PSI:Biology-Materials Repository:* Browse the PSI:Biology-Materials Repository by center. The results will list all plasmids from the center.

### Plasmid information

For a researcher to use a plasmid from DNASU effectively and easily, the clone details were overhauled to provide detailed, well-annotated information about each plasmid on a single easy-to-navigate page that can be accessed directly from the search results. Plasmid information is now organized into three tabs containing information about the gene insert, the vector and annotations.

#### Gene details tab

The gene detail tab contains all information about the gene insert. This includes three main sections:

1. ***Synopsis*.** Information including gene name, institutions and researchers who deposited the plasmid, and publication information provided by the depositing scientist or researchers who previously requested the plasmid from DNASU.
2. ***Sequence data*.** Nucleotide sequence information, including the entire insert sequence (open reading frame highlighted in gray) and the flanking sequences (when available). This section also provides on-the-fly protein translation and information about any mutations in the nucleotide or protein sequence.
3. ***Plasmid handling*.** The antibiotic selection required to grow the plasmid. For the majority of PSI plasmids, protein expression information, including the recommended expression host, the expression and purification results (if already expressed by the PSI center), and links to Target Track, which contains the experimental protocols for expression and purification are also listed.

Also on this tab is a box with information about price, availability, Material Transfer Agreement (MTA) details and a button to request the plasmid.

#### Vector details tab

The plasmid’s vector information, found on the second tab, lists institutions and researchers who created the vector, the full sequence and the map that was provided by the depositing scientist. If available, a link to the empty vector, which can be used as a control in an experiment or for cloning, is displayed.

**Figure 2.**
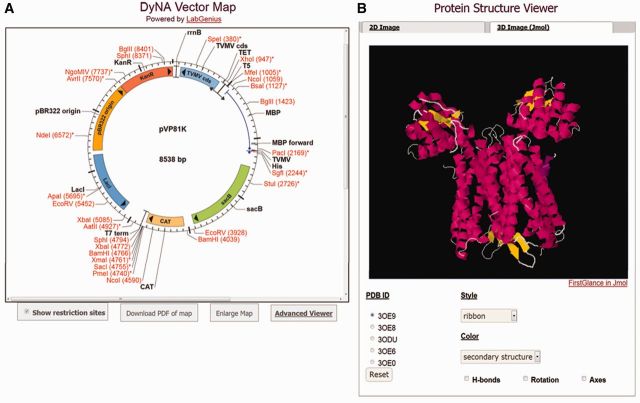
**(A**) The Dynamic (DyNA) Vector Map, a widget created by LabGenius. (**B**) The PDB Structure viewer shows both the 2D and a dynamic 3D view of the protein structure. Selecting different PDB IDs will automatically switch between structures available for that gene.

The new Dynamic (DyNA) Vector Map widget was created in collaboration with LabGenius (Figure 2A). The map displays vector features, and scrolling over a feature reveals a pop-up containing the start and stop positions of the feature and its description. These were manually annotated by the repository to ensure accurate display of features. Clicking ‘Show restriction sites’ maps selected restriction sites onto the vector, with unique sites indicated by an asterisk. The DyNA Map can be downloaded with or without the restriction sites and includes a table of all features and restriction sites along with their positions. The Advanced Viewer links out to the LabGenius website, which provides additional free tools to analyze the vector sequence further, design primers for cloning or sequencing and perform virtual restriction digests.

#### Annotations tab

The goal of the ever-expanding annotations tab is to provide links to additional information about the gene insert within the plasmid. These annotations can be roughly categorized into nucleotide, protein and experimental annotations [summarized in Table 3 (20–24)]. For plasmids that were used to determine the structure of a protein, we created a new structure viewer that presents a 2D image of the structure and a Jmol-enabled applet to explore the 3D structure (Figure 2B).

**Table 3.** A selection of the annotation websites that are linked to from the plasmid details page

Annotation Type	Database name	Content description	URL
Nucleotide	GenBank	Protein or nucleotide GI or GenBank Accession number linked to either the NCBI protein or nucleotide database.	http://www.ncbi.nlm.nih.gov
	FLEX	Links to the internal sample tracking database FLEX, which contains gene sequence and linker information.	http://flex.asu.edu/FLEX/
Protein	SMART	A database that allows for the identification and annotation of genetically mobile domains and the analysis of domain architectures.	http://smart.embl-heidelberg.de/
	Structural Biology Knowledgebase (SBKB)	A portal to research data and other PSI resources. Includes Hubs that link to discoveries, target information, structural information, methods and models.	http://sbkb.org
	Uniprot	The Universal Protein Resource provides protein sequence and functional information.	http://www.uniprot.org
	Protein Databank (PDB)	An information portal for protein structures.	http://www.rscb.org
	TOPSAN	A Wiki focused on sharing information about protein structures.	http://www.topsan.org
	Labome	A laboratory resource site contains links to reagent resources, reviews and methods.	http://www.labome.com
Experimental	Target Track	Provides information on the experimental progress and status of targets selected for structural determination by the PSI.	http://www.sbkb.org/tt/
	SBKB technology portal	Contains articles highlighting the latest technologies and methods used in high-throughput structural biology efforts.	http://technology.sbkb.org
	SBKB featured articles	Articles highlighting specific structures, technologies or systems presented by the SBKB in collaboration with nature	http://sbkb.org/update/research/
	SBKB technical highlight		
	SBKB featured system		
	PSI center websites	Contains additional information about plasmids, proteins, cloning and methods	Various

### Plasmid distribution

#### Requesting plasmids

Researchers can request plasmids and plasmid collections directly through the DNASU website by registering for a free account, finding the plasmid(s) of interest and adding the plasmid(s) to the cart. All plasmids are stored as bacterial glycerol stocks in 2D-barcoded tubes in a Brooks BioStore freezer storage and retrieval system, allowing fast robotic cherry picking of samples as soon as an order is placed. In order to offset the costs of preparing the plasmid for shipment and to ensure the viability of the repository in the future, we have implemented a nominal recharge fee with discounts provided for full 96-well plates of plasmids and special predetermined collections to facilitate high-throughput studies. Over 4200 orders containing 200 000 plasmids have been shipped worldwide from DNASU to researchers in 47 states and 40 countries.

When researchers request plasmids, they typically require them for an experiment urgently. With the goal of eliminating the delays caused by waiting for the MTA to be reviewed and signed, we created an Expedited MTA Network [described in detail in (10)]. With the MTA terms agreed to in advance and institutional signatures already in place, plasmids can be sent immediately after an order is electronically placed. With over 260 Member institutions, half of all registered users are members of Expedited MTA Network institutions and >60% of all orders from DNASU benefit from being part of this growing Network. In the past 4 years, this system has been improved by providing Technology Transfer Officials (TTO) at member institutions two new reporting options. First, each TTO automatically receives an email, which includes information about who placed the order and what was ordered, at the time of shipping. Second, the TTOs have 24/7 secure access to a reporting feature on DNASU listing all orders and order details from their institution. These features, requested by Member institutions, allow TTOs to keep track of materials entering their institutions to ensure that their internal regulations are followed.

DNASU is dedicated to assisting researchers. To fulfill that goal, all questions about DNASU can be directed to a PhD-level scientist at a dedicated service email address dnasuhelp@asu.edu, which will be answered within 24 h.

#### Depositing plasmids

DNASU openly encourages researchers to deposit plasmids in the repository. Depositors will be credited for sharing their materials and will benefit from having a secure archive site by being alleviated from the burden of storage, maintenance and distribution of their plasmids. The general research community benefits from having plasmids rapidly available from a central source. There is a standardized three-step process to deposit plasmids. First, we work with the depositing institution to establish the Depositor Agreement, the legal agreement that sets forth the terms by which DNASU can distribute plasmids deposited by that institution. This step is often fast if there is already a Depositor Agreement from that institution on record. Second, the researcher will provide information about each plasmid in an Excel spreadsheet. This is the information that will be presented on the Plasmid Details pages on the DNASU website. Finally, they will send the plasmid sample(s). We will transform the DNA and sequence the gene insert with an end-read to confirm its identity before releasing it publicly in DNASU. Researchers interested in submitting plasmids to DNASU can find details on the website http://dnasu.org/DNASU/Submission.jsp and the PhD-level Scientific Liaison will facilitate each step of the submission process.

## FUTURE DIRECTIONS

As DNASU continues to grow, we maintain the mission of storing and distributing plasmids while expanding the information for each plasmid available through DNASU. Toward this end, we plan to expand the collection by partnering with researchers to deposit plasmid collections with DNASU. To ensure accurate and up-to-date plasmid information, we are implementing an automated gene annotation framework to compare gene insert sequences with the most recent reference sequence in NCBI and Ensembl. In addition, annotations will be expanded by providing pathway and protein–protein interaction information for genes and visually displaying these in interactive browse features, which will allow researchers to search and build customized clone sets based on gene functions of interest. Finally, by collaborating with laboratory and research management websites, such as LabGuru and linking plasmids to their publications and additional experimental resources, we hope to integrate the plasmids in DNASU into researchers’ experimental designs seamlessly. With the ever-increasing time and monetary constraints within laboratories, we expect that DNASU and other plasmid repositories will continue to provide valuable resources to facilitate biological research.

## FUNDING

The National Institute of General Medical Sciences [U01 GM098912]; Virginia G. Piper Charitable Trust. Funding for open access charge: [NIGMS: U01 GM098912].

*Conflict of interest statement*. None declared.
